# 

**Published:** 1894-06-16

**Authors:** 


CONTENTS.

**Reactionary Medicine** - 221
**Around the Hospitals** - 222
**On Modern Progress in Ophthalmic Medicine and Surgery.** By Robert Brudenell Carter, F.R.C.S. - 223
**The First Century of English Medical Literature.** - 224
**The Study of Man.** VI.—First Traces and Their Interpretation - 225
**Medical Progress and Hospital Clinics—**
Observations Regarding Chronic Heart Disease Complicating Pregnancy and Labour ((*continued*) - 229
Acute Intussusception. II. - 231
Medical Progress.—Fevers and Infectious Diseases - 232
Progress in Surgery.—Surgery of the Intestines (*continued*) - 234
New Appliances and Things Medical - 234
**The Field of Science** - 226
**Annotations.**—Old-fashioned Practice—Should Doctors Trade?—The Prevention of Suicide - 227
**Notes and News** - 228
**St. Thomas's Hospital Medical School** - 235
**Editor's Letter-Box.** Eucalyptus Oil - 236
**The Institutional Workshop—**
Hospital Administration.—Birmingham Hospital Saturday Fund - 237
Hospital Festivals and Meetings - 239
**The Mansion House Hospital Sunday Conversazione** - 239
**Scraps and Gleanings** - 240
**The Student of Medicine** - 242

EXTRA SUPPLEMENT.—THE NURSING MIRROR.

**News from the Nursing World.**—Practical Charity—Dissatisfied—Nurses at Peterborough—Long Nights—Proposed Increase of Nursing Staff—An Alarming Storm—The "Registered" Nurses' Society—A Pleasant Afternoon—Prince Alfred Hospital, Sydney—Melbourne—Nurses at Philadelphia—"A Dreadful Eruption"—Mutual Benefit—Graduates at Brooklyn, U.S.A.—Pet Economies—Short Items - c
**On General Nursing.** XVI.—Rheumatic Gout - ciii
**The Mechanism of Movement.** III.—Structure of Bone - civ
**The Royal British Nurses' Association—Appointment** cv
**Poplar Hospital for Accidents** - cvi
**Nursing in Germany** - cvii
**Our Examinations—Where to Go** - cviii
**Nurses for Civilians in India** - cviii
**The Muses' Looking-Glass** - cix
**Everybody's Opinion** - cx
**Royal Asylum, Morningside, Edinburgh** - cx
**Minor Appointments—Presentations** - cx

